# State-to-state approach in hypersonic thermochemical nonequilibrium flow simulations: a review

**DOI:** 10.3389/fchem.2026.1869326

**Published:** 2026-07-01

**Authors:** Xianying Chu, Wei Zhao, Hongjun Zhang, Jingying Wang, Song Fu, Shiyue Zhang, Chunhian Lee

**Affiliations:** 1 School of Aerospace Engineering, Tsinghua University, Beijing, China; 2 National Key Laboratory of Aerospace Flight Technology, Beijing, China; 3 Beijing Aerospace Technology Institute, Beijing, China; 4 School of Nuclear Science, Energy and Power Engineering, Shandong University, Jinan, China; 5 Shenzhen Research Institute of Shandong University, Shenzhen, China; 6 School of Aeronautic Science and Engineering, Beihang University, Beijing, China

**Keywords:** computational fluid dynamics, hypersonic flows, rate coefficients, state-to-state model, thermochemical nonequilibrium

## Abstract

Thermochemical nonequilibrium flows play a critical role in shaping the aerothermal environment of hypersonic vehicles and therefore strongly influence the analysis and design of thermal protection systems (TPS). Traditional multi-temperature models often fail to accurately represent non-Boltzmann population distributions and detailed vibration-dissociation coupling, whereas state-to-state (StS) models provide a more physically rigorous description by directly resolving transitions between internal energy levels. This review summarizes recent advances in StS modeling for hypersonic thermochemical nonequilibrium flows, including elementary rate-coefficient determination, benchmark applications in canonical nonequilibrium flows, and current extension strategies based on reduced-order modeling, coarse graining, and machine-learning-assisted acceleration. The reviewed literature shows that the reliability of StS predictions depends strongly on the availability, accuracy, and consistency of elementary kinetic data, while their broader engineering use remains limited by the enormous number of internal states and elementary processes, severe equation stiffness, incomplete kinetic databases, and insufficient experimental data for robust validation. Overall, this review clarifies the recent progress, key bottlenecks, and promising future directions of StS modeling in hypersonic nonequilibrium flows.

## 1 Introduction

Hypersonic flight technology plays a critical role in the development and utilization of space resources (Bertin and Cummings, 2003). Hypersonic vehicles typically traverse or re-enter the Earth atmosphere (30-70 km) at extremely high speeds, with Mach numbers generally exceeding 5 (Anderson Jr, 2006; Bose et al., 2013; Olynick et al., 1999). During this process, a strong shock forms ahead of the vehicle, causing the freestream air to undergo intense compression. This compression converts a significant portion of the kinetic energy into thermal energy, raising the gas temperature to several thousand or even tens of thousands of kelvins and giving rise to high-temperature gas effects. The internal energy modes of atmospheric molecules become highly excited, deviating from the Boltzmann distribution and in some cases leading to thermal radiation. Meanwhile, atmospheric species undergo complex chemical reactions, including dissociation, exchange, and ionization. Strong coupling exists between internal energy excitation and chemical reactions; for example, vibrational excitation facilitates molecular dissociation, whereas dissociation leads to the depletion of vibrational energy. The characteristic timescales of internal energy excitation and chemical reactions in high-temperature air are comparable to the flow timescales, resulting in thermochemical nonequilibrium.

Owing to technological limitations, it is highly challenging to reproduce hypersonic high-temperature thermochemical nonequilibrium flows in ground-based high-enthalpy facilities (Bertin and Cummings, 2006; Gu and Olivier, 2020; Hirschel, 2005). Computational fluid dynamics (CFD) has therefore become an important tool for investigating such flows, and the accuracy of CFD predictions of high-temperature gas effects depends fundamentally on whether the physical models can accurately represent the thermochemical nonequilibrium processes in high-temperature air.

Currently, simulations of hypersonic thermochemical nonequilibrium flows primarily rely on multi-temperature models. The fundamental assumption of these models is that the internal energy levels of atmospheric species satisfy the Boltzmann distributions characterized by their respective temperatures. Based on the original Navier-Stokes equations, additional energy transport equations are introduced to determine the temperatures of the internal energy modes and the energy exchange among them. The rates of chemical reactions, such as dissociation and ionization, are governed by an empirical temperature, typically defined as a power-law average of the temperatures associated with different internal energy modes. Researchers such as Lee (Lee, 1984), Candler (Candler and Park, 1988), and Kim (Kim and Boyd, 2013) have proposed three-temperature models, considering translational-rotational, vibrational, and electron temperatures separately. These multi-temperature models are essentially empirical reduced-order models, characterized by their simplicity and suitability for solving multidimensional problems. They remain the most widely used models for studying thermochemical nonequilibrium effects in the high-temperature atmospheric flows encountered by hypersonic vehicles. Well-known solvers, including DPLR (Wright et al., 1998), LAURA (Sutton and Gnoffo, 1998), CFD++ (Ramjatan et al., 2017), LeMANS (Scalabrin, 2007), US3D (Nompelis and Candler, 2014), FUN3D (Gnoffo et al., 2013), and Ansys Fluent (Xia et al., 2024), all employ the Park two-temperature model. In fact, other thermochemical nonequilibrium models developed during the same period share similar characteristics with multi-temperature models. For instance, the Macheret-Fridman model (Macheret et al., 1994) and the CVDV model (Marrone and Treanor, 1963) also assume that the internal energy levels of high-temperature atmospheric species follow Boltzmann distributions, differing from multi-temperature models primarily in their treatment of chemical reaction rates.

However, recent studies have shown that the widely used multi-temperature models fail to capture the non-Boltzmann distribution characteristics of the internal energy levels in real high-temperature air. As a result, they cannot accurately simulate critical problems such as hypersonic shock/boundary layer interactions, electron number density in re-entry vehicle flows, and nonequilibrium flows in hypersonic nozzles (Hao et al., 2017a; 2017b; 2017c).

To overcome these limitations, state-to-state (StS) models have been developed, providing a more detailed description by directly resolving transitions between the energy levels of atmospheric species. Macroscopic phenomena are the statistical results of microscopic physical processes. Therefore, understanding thermochemical nonequilibrium flows requires analysis of microscopic processes to reveal the underlying mechanisms. At the microscopic level, macroscopic thermodynamic processes and chemical reactions can ultimately be decomposed into elementary excitation and de-excitation processes involving all energy levels. Based on quantum mechanics, the StS model treats each energy level of the internal energy modes of atmospheric species as a “pseudo-component” and solves the master equations governing the elementary excitation and de-excitation processes for each level. This approach provides detailed energy level distribution data and directly captures chemical reactions at each energy level, thereby avoiding the empirical vibration-dissociation coupling treatments used in multi-temperature models (Nagnibeda and Kustova, 2009). Thus, StS models are regarded as the most accurate approach for modeling thermochemical nonequilibrium processes in high-temperature gases. The accuracy of StS simulations strongly depends on the accuracy of the rate coefficients for elementary processes. However, the extensive number of energy levels in atmospheric molecules and atoms, together with the corresponding nonequilibrium elementary processes, results in computational demands that exceed the capabilities of current hardware. In practice, hypersonic research primarily aims to accurately predict macroscopic aerodynamic characteristics under high-temperature conditions rather than detailed microscopic energy level distributions. As a result, StS models are generally limited to zero-dimensional problems or to specific flow configurations, such as post shock flows, boundary layers, and quasi-one-dimensional nonequilibrium nozzle flows. To extend the application of StS methods to thermochemical nonequilibrium modeling while reducing the computational cost, two main approaches have been adopted. The first involves modifying multi-temperature models based on the results of StS simulations. The second focuses on reducing the complexity of StS simulations through energy levels grouping, with coarse-grained models receiving increasing attention.

This review provides a comprehensive overview of StS methods and their applications to hypersonic thermochemical nonequilibrium flows. Although electronic-state, rovibrational, and surface-coupled StS extensions are also highly relevant to hypersonic nonequilibrium flows, especially for radiation, rarefied-gas dynamics, and gas-surface interaction problems, the present review primarily focuses on vibrational StS modeling and its use in thermochemical nonequilibrium CFD, where the current literature is most developed and most directly connected to reduced-order modeling and machine-learning-assisted acceleration. Section 2 introduces the fundamental concepts of elementary processes in StS simulations and reviews the methods used to determine the rate coefficients of these processes. Section 3 focuses on the recent progress in the application of StS methods to high-enthalpy nonequilibrium flows, including their use in low-dimensional problems and the development of reduced-order models aimed at extending these methods to higher-dimensional flows. Section 4 summarizes the application of machine learning to StS calculations. Section 5 summarizes the key findings on StS simulations in hypersonic nonequilibrium flows and provides a forward-looking discussion of the remaining challenges.

## 2 State-to-state reaction kinetics

### 2.1 Elementary processes

StS models can be classified into electronic, rovibrational, and vibrational models based on the granularity of internal energy levels. Electronic StS models (Du et al., 2022; Hong et al., 2020), often formulated within collisional-radiative frameworks, are particularly important for radiation prediction in high-enthalpy re-entry flows. In such models, nonequilibrium populations of electronically excited states and their coupling with radiative transitions play a central role in determining emission- and ionization-related observables. However, compared with vibrational StS modeling, electronic-state modeling involves much larger state spaces and stronger coupling with radiation transport, and therefore constitutes a specialized topic in its own right. Rovibrational StS models further increase the resolution of nonequilibrium kinetics by resolving both vibrational and rotational energy levels. Such models are attractive when rotational nonequilibrium or state-specific rotational effects are expected to influence relaxation or dissociation. However, the associated state-space growth is substantial, which strongly limits their applicability in practical CFD. As a result, rovibrational StS approaches are still used mainly in simplified systems, benchmark studies, or high-fidelity reference calculations (Schwartzentruber et al., 2018).

Among these categories, vibrational StS models have received the most attention (Wang H. et al., 2023). These models focus on transitions between vibrational energy levels within the ground electronic state of molecules, as well as on chemical reactions associated with each vibrational level. Their current prominence is closely related to the balance they offer between physical fidelity and computational feasibility, especially in thermochemical nonequilibrium CFD. It should also be noted that the broader application of StS models depends not only on the level structure being resolved, but also on several related issues. In particular, the reliability of high-fidelity state-specific rate coefficients is strongly influenced by the quality of the underlying potential energy surfaces (PESs), especially in QCT-, QC-, and QM-based calculations. In addition, DSMC-StS coupling is important for rarefied and transitional nonequilibrium flows, whereas wall boundary conditions and surface chemistry are essential for extending StS modeling to catalytic recombination, gas-surface interaction, and ablation-related problems. Although these topics are highly important and are therefore briefly addressed here, the main focus of the present review remains on vibrational StS modeling in hypersonic applications.

The elementary processes in vibrational StS models typically include vibrational-vibrational-translational (V-V-T) bound-bound transitions induced by molecular impacts, vibrational-translational (V-T) bound-bound transitions induced by atomic impacts, and vibrational-dissociation (V-D) bound-free transitions occurring in both two interaction types. Considering these types of elementary processes in vibrational StS models, the master equation governing the vibrational energy level distribution of the A_2_ molecule can be written as follows (Hao et al., 2017c):

$$\frac{\partial \left[A_{2}\left(i\right)\right]}{\partial t}={\left\{\frac{\partial \left[A_{2}\left(i\right)\right]}{\partial t}\right\}}_{V–V–T}+{\left\{\frac{\partial \left[A_{2}\left(i\right)\right]}{\partial t}\right\}}_{V–T}+{\left\{\frac{\partial \left[A_{2}\left(i\right)\right]}{\partial t}\right\}}_{V–D}$$

where

$$\begin{array}{l} {\left\{\frac{\partial \left[A_{2}\left(i\right)\right]}{\partial t}\right\}}_{V–V–T}=\sum_{m}\sum_{n}\sum_{j}k_{V–V–T}\left(m,n\to i,j\right)\left[A_{2}\left(m\right)\right]\left[A_{2}\left(n\right)\right]\\ -\sum_{m}\sum_{n}\sum_{j}k_{V–V–T}\left(i,j\to m,n\right)\left[A_{2}\left(i\right)\right]\left[A_{2}\left(j\right)\right] \end{array}$$

$${\left\{\frac{\partial \left[A_{2}\left(i\right)\right]}{\partial t}\right\}}_{V–T}=\sum_{j\neq i}\left\{k_{V–T}\left(j\to i\right)\left[A_{2}\left(j\right)\right]\left[A\right]-k_{V–T}\left(i\to j\right)\left[A_{2}\left(i\right)\right]\left[A\right]\right\}$$

$$\begin{array}{l} {\left\{\frac{\partial \left[A_{2}\left(i\right)\right]}{\partial t}\right\}}_{V–D}=\left\{k_{V–D}^{A_{2}}\left(c\to i\right){\left[A\right]}^{2}\left[A_{2}\right]-k_{V–D}^{A_{2}}\left(i\to c\right)\left[A_{2}\left(i\right)\right]\left[A_{2}\right]\right\}\\ +\left\{k_{V–D}^{A}\left(c\to i\right){\left[A\right]}^{3}-k_{V–D}^{A}\left(i\to c\right)\left[A_{2}\left(i\right)\right]\left[A\right]\right\} \end{array}$$

In these expressions, [A] denotes the number density of species A. *k*_V-V-T_ (*i*,*j* → *m*,*n*) and *k*_V-T_ (*i* → *j*) denote the transition rate coefficients for V-V-T and V-T processes, respectively. Similarly, *k*_V-D_ (*i* → *c*) and *k*_V-D_ (*c* → *i*) represent the dissociation and recombination rate coefficients associated with vibrational level *i*, respectively, where the superscripts indicating the collision partner. For atomic A, the corresponding equations can be written in a similar form as follows:

$$\frac{\partial \left[A\right]}{\partial t}=\sum_{i}\left[-2{\left(\frac{\partial \left[A_{2}\left(i\right)\right]}{\partial t}\right)}_{V–D}\right]$$

The master equation can be directly coupled with the governing equations of compressible flow to track the spatial evolution of the population at each vibrational level of A_2_.

### 2.2 Calculation of elementary process rate coefficients

The fidelity of StS simulations depends on the accuracy of the rates coefficients for elementary process. However, owing to the large number of elementary processes, experimental measurements can cover only a small fraction of them, and such experiments are often limited by technical challenges and measurement uncertainties. As a result, the rates coefficients for elementary processes are predominantly determined using theoretical models or numerical calculations.

For vibrational StS models, early studies primarily relied on empirical or semi-empirical approaches to determine rate coefficients. Under the assumption of single-quantum vibrational transitions, Landau and Teller (Vincenti et al., 1965) first proposed a linear semi-empirical expression for the rate coefficients of V-T process. This model has been widely used in numerical simulations of hypersonic thermochemical nonequilibrium flows. However, under high-temperature conditions, the assumptions of harmonic oscillator and Boltzmann distributed populations are no longer valid, causing the vibrational relaxation times predicted by the Landau-Teller model being significantly larger than those measured experimentally (Macheret et al., 2002). Schwartz et al. (Schwartz et al., 1952; Sharma et al., 1992) theoretically investigated collinear collisions between non-rotational anharmonic oscillators and spherically symmetric structureless particles. Using semiclassical first-order perturbation theory, they developed the Schwartz-Slawsky-Herzfeld (SSH) model. This model estimates the rate coefficients for higher energy levels by scaling those for lower energy levels, which leads to substantial errors in collision probabilities at high temperatures and may even yield nonphysical transition probabilities (Adamovich et al., 1995a). Because the SSH model is based on first-order perturbation theory, it accounts only for single-quantum vibrational transitions and cannot be readily extended to multi-quantum transitions. Some studies have combined the SSH model with experimental measurements to determine rate coefficients (Armenise and Kustova, 2013), but the scarcity of experimental data limits its applicability to specific types of transition processes.

**FIGURE 1:**
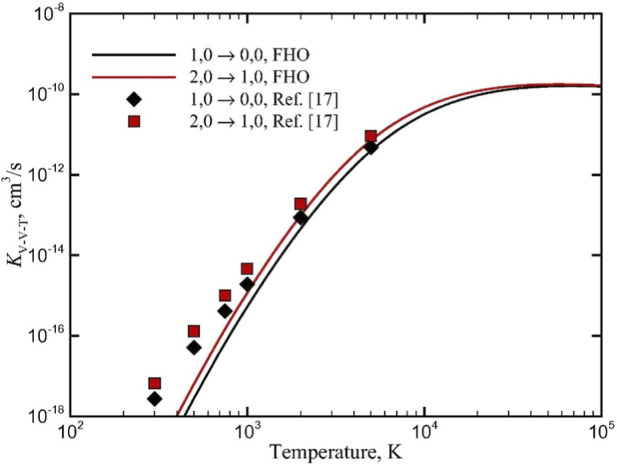
Single quantum transition rate coefficients for O_2_-O_2_ collisions (Hao et al., 2017c). Ref [17] in the figure refers to the semiclassical trajectory calculations by Billing and Kolesnick (1992) in this review.

**FIGURE 2:**
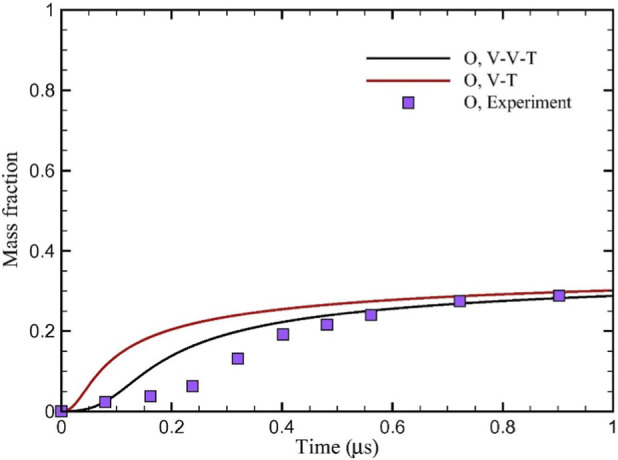
Post shock mass fraction profiles of atomic O predicted with and without V–V transitions (Hao et al., 2017c).

To address the limitations of the SSH model, Adamovich et al. (Adamovich et al., 1995a; 1995b; 1998) proposed a semiclassical, semi-perturbative analytical model known as the Forced Harmonic Oscillator (FHO) model. This model can be used to calculate transition rate coefficients for V-V-T, V-T, and V-D processes and can be extended to multi-quantum transitions. The transition from SSH model to the FHO model reflects an effort to improve the physical description of multiquantum transitions and dissociation while retaining analytical tractability, and comparisons with trajectory-based calculations have played a key role in assessing this improvement. Lino da Silva et al. (Silva et al., 2007; 2012) used the FHO model to reduce V-V-T processes into V-T processes by neglecting pure V-V transitions. They derived V-T and V-D transition rates for N_2_+N_2_ and O_2_+O_2_ collisions, applicable to temperatures up to 10^5^ K. Hao et al. (2017c) expanded the database of V-V-T rate coefficients for O_2_+O_2_ collisions using the FHO model (Figure 1) and shown that neglecting V-V processes shortens the relaxation and incubation times, thereby leading to higher dissociation rates under strongly nonequilibrium conditions (Figure 2). Gu et al. (2022) further improved StS models for air species by calculating V-V-T rate coefficients for N_2_+N_2_, N_2_+O_2_, and O_2_+O_2_ collisions using the FHO model. However, the simplified assumptions regarding the potential energy function in the FHO model, together with its reliance on phenomenological parameters, mean that the data generated by the model still require further validation.

Because they rely on empirical parameters, the rate coefficients obtained from the aforementioned empirical or semi-empirical models can no longer meet the increasing demands of high-precision simulations. As a result, methods for deriving elementary rate coefficients in vibrational StS models through molecular dynamics simulations have attracted significant attention in recent years. The most accurate method for kinetic simulations is the quantum mechanical (QM) approach (Kosloff, 1988), but its computational cost is extremely high. The computational cost increases rapidly with the total collision energy, which limits its application to low rovibrational energy levels and makes it unsuitable for high-temperature conditions. To reduce the computational cost while maintaining acceptable accuracy, the quasi-classical trajectory (QCT) method (Esposito and Capitelli, 2007; Karplus et al., 1965) and the quantum-classical (QC) hybrid method (Billing, 1984; 2003) have been developed. However, semiclassical or classical methods may show systematic deviations from quantum mechanical (QM) results at low collision energies, near threshold processes, or where quantum effects are significant. The QCT method uses Monte Carlo sampling on the potential energy surface (PES) to compute molecular trajectories and determine transition rate coefficients for arbitrary vibrational energy levels. Although highly efficient, this method cannot accurately capture quantum effects. Nevertheless, under high-temperature conditions in which quantum effects become negligible, the classical mechanical treatment adopted in QCT remains reliable. In contrast, the QC method incorporates quantum corrections, and achieves slightly higher accuracy than QCT, but at a significantly greater computational cost. Consequently, QCT is widely used to calculate elementary rate coefficients in high-temperature StS models for air species. Recent developments in mixed quantum-classical formulations further indicate continued efforts to improve the treatment of highly excited vibrational states while mitigating the prohibitive cost of fully quantum approaches (Yang et al., 2025). Overall, the progression from SSH to FHO, QCT, QC, and QM reflects a systematic increase in physical fidelity, accompanied by a corresponding increase in computational cost, as shown in Table 1. Accordingly, no single method is universally optimal: empirical and semi-empirical models remain useful for low-cost engineering-oriented approximations, FHO-type models provide a practical intermediate-fidelity route for state-specific database construction, and trajectory- or quantum-based methods are more appropriate when high-fidelity kinetic data are required for detailed StS simulations or model validation.

**TABLE 1:** Comparison of major methods for determining elementary rate coefficients in StS modeling.

Method	Physical fidelity	Computational cost	Main limitations	Most appropriate use cases
SSH	Low to moderate	Low	Limited to single-quantum transitions; may yield large errors at high temperature; poor treatment of strongly nonequilibrium and multiquantum processes	Low-cost database construction; qualitative trend analysis
FHO	Moderate	Low to moderate	Depends on simplified interaction potentials and phenomenological parameters; accuracy still requires validation	Intermediate-fidelity state-specific databases; reduced-order model development
QCT	Moderate to high	Moderate to high	Cannot fully capture quantum effects, especially at low energies or for threshold-sensitive processes	High-temperature air kinetics; database generation for StS simulations
QC	High	High	More computationally expensive; still less general than QCT	Benchmark-quality datasets; systems where quantum effects remain important
QM	Very high	Very high to prohibitive	Computationally prohibitive for large systems, high energies, or broad temperature ranges	Small systems; low-energy collisions; benchmark validation of reduced models

**FIGURE 3:**
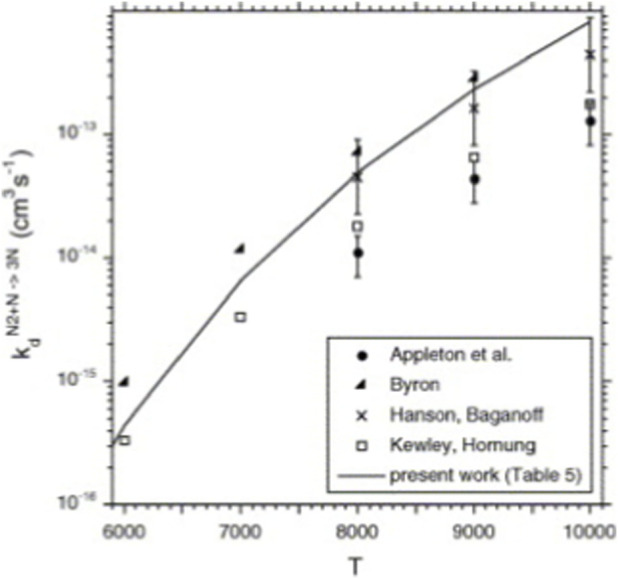
Experimental measurements (points) and Esposito work (Esposito et al., 2006) (line) global dissociation rate coefficients versus *T* for the process N_2_+N→3N.

Esposito et al. (Esposito and Capitelli, 2007; Esposito et al., 2008), Andrienko et al. (Andrienko and Boyd, 2015b; 2015c; 2016), and Kulakhmetov (Kulakhmetov, 2016) calculated V-T and V-D transition rate coefficients for O_2_+O collisions. Esposito et al. (2006) and Jaffe et al. (Jaffe et al., 2009; 2010) calculated V-T and V-D transition rate coefficients for N_2_+N and validated the V-D rate coefficients by comparison with experimental measurements. As shown in Figure 3, the predicted data fall within a reasonable error range. Jaffe et al. (Jaffe et al., 2009; 2010) and Bender (Bender, 2016) also calculated V-D transition rate coefficients for N_2_+N_2_ collisions. These studies provided data applicable over a broad temperature range, with an upper limit of 10^5^ K. To supplement the rate coefficient database for the 300-5000 K range, Hong et al. (Hong et al., 2021; 2022; 2023) employed the QC method to calculate vibrational rate coefficients for air collision systems, including N_2_+N_2_, O_2_+O_2_, and N_2_+O. Lopez and da Silva (Lopez and Lino Da Silva, 2014) systematically compiled state-to-state rate constants reported in the literature and established the STELLAR database, which provides an important basis for assembling reaction datasets for air StS modeling. On this basis, Hong (2022) further summarized the elementary rate coefficients adopted for a five-species air system, as listed in Table 2. Specifically, the rate coefficients for VT1, VT4, and VT7 were obtained from calculations based on the Forced Harmonic Oscillator (FHO) model (Kerner, 1958), whereas those for VT2 and VT5 were taken from the Quasi-Classical Trajectory (QCT) results reported by Esposito et al. (Esposito et al., 2006; 2008). For dissociation and recombination processes, DR1, DR3, and DR5 were derived from the extended FHO model (Macheret and Adamovich, 2000), while DR2 and DR4 were adopted from the QCT data (Esposito et al., 2006; 2008). For excitation processes, the rate coefficients of EX1 and EX2 were taken from the QCT calculations (Bose and Candler, 1996; 1997), respectively. In addition, VVT1 and VVT2 were calculated by Hong using the FHO approach, VT3, VT6, and VT8 were estimated using the Landau–Teller (LT) model, and the rate coefficient of DR6 was supplemented on the basis of the CVDV method. Overall, this compilation highlights that current state-specific air databases are not derived from a single consistent theoretical framework, but rather assembled from heterogeneous sources with different levels of physical fidelity. This lack of consistency should be recognized as an important source of uncertainty when such databases are used in high-fidelity StS simulations of hypersonic nonequilibrium flows.

**TABLE 2:** Representative sources of elementary rate coefficients used in a five-species air StS database (Hong, 2022).

Reaction	Method	Reactions	Method
VT1: N2(v)+M<=>N2(w)+M, M = O2,NO	FHO	DR1: N2(v)+M<=>2N + M, M = N2,O2,NO	FHO
VT2: N2(v)+N<=>N2(w)+N	QCT	DR2: N2(v)+M<=>2N + M, M = N,O	QCT
VT3: N2(v)+O<=>N2(w)+O	LT	DR3: O2(v)+M<=>2O + M, M = N2,O2,NO	FHO
VT4: O2(v)+M<=>O2(w)+M, M = N2,NO	FHO	DR4: O2(v)+M<=>2O + M, M = O,N	QCT
VT5: O2(v)+O<=>O2(w)+O	QCT	DR5: NO(v)+M<=>N + O + M, M = N2,O2	FHO
VT6: O2(v)+N<=>O2(w)+N	LT	DR6: NO(v)+M<=>N + O + M, M = N,O,NO	CVDV
VT7: NO(v)+M<=>NO(w)+M, M = N2,O2,NO	FHO	EX1: N2(v)+O<=>NO(w)+N	QCT
VT8: NO(v)+M<=>NO(w)+M, M = N,O	LT	EX2: O2(v)+N<=>NO(w)+O	QCT
VVT1: O2(v)+O2(w)≤>O2 (v’)+O2 (w’)	FHO	VVT2: N2(v)+N2(w)≤>N2 (v’)+N2 (w’)	FHO

## 3 High-fidelity state-to-state simulation and modeling in thermochemical nonequilibrium flow

### 3.1 State-to-state simulation in high-enthalpy nonequilibrium flow

StS models can accurately simulate transition processes between molecular energy levels (Capitelli et al., 1997; Silva et al., 2007), offering significant advantages for investigating complex effects such as vibrational relaxation, dissociation, and recombination in high-temperature gases. However, such simulations involve a large number of reactions and depend strongly on the microscopic models and rate coefficients employed. As a result, coupling StS simulations with three-dimensional flow solvers remains highly challenging. Early StS studies primarily focused on zero-dimensional heat baths (Colonna et al., 2006; Duan et al., 2024; Xu et al., 2014), one-dimensional post shock wave flows (Adamovich et al., 1995a; 1995b; Bruno et al., 2002; Gimelshein et al., 2022; Gu et al., 2022; Hong et al., 2019; Su et al., 2017), boundary layers flows (Armenise and Capitelli, 2005; Armenise et al., 1995; 1996; 1998), and quasi-one-dimensional nonequilibrium nozzle flows (Colonna et al., 1998; 1999; 2003). These low-dimensional studies provide the foundation for extending StS methods to multi-dimensional flows, while also supplying results that can be used to refine existing macroscopic models and develop new ones. As StS models continues to advance, they are increasingly being applied to two-dimensional and axisymmetric flows. This progress has opened new avenues for the application of StS models to specific problems, such as shock/boundary layer interactions and wall ablation phenomena, thereby broadening their scope in research on high-enthalpy flows.

In zero-dimensional StS simulations, Colonna et al. (2006) qualitatively investigated boundary layer, nozzle, and shock flows behavior in the N_2_+N gas system. They derived analytical solutions to the master equations under specific conditions and compared the results with those obtained from a one-dimensional coupled flow model. This comparison demonstrated the potential of the homogeneous StS kinetic model for use in macroscopic CFD simulations. Xu et al. (2014) investigated thermochemical nonequilibrium in the N_2_+N gas mixture, identifying the V-T process as a key factor governing vibrational energy distribution and revealing distinct transition characteristics under dissociation-dominated and recombination-dominated conditions. Duan et al. (2024) investigated the CO_2_ system by considering 201 energy levels among its three vibrational modes: symmetric stretching, bending, and asymmetric stretching. The results showed that V-T processes dominate in the CO_2_ system, with the bending mode exhibiting the highest excitation rate. Additionally, higher isothermal heat bath temperatures were found to increase vibrational transition rates. However, the study did not consider the coupling between chemical reactions and the three vibrational modes of CO_2_.

StS models have gradually been extended to more complex flow configurations, including post-shock flows, boundary layers, and quasi-one-dimensional nozzle flows. In post shock flow studies, Adamovich et al. (Adamovich et al., 1995a; 1995b) investigated vibrational relaxation and dissociation behind strong shocks using a corrected FHO model in N_2_ and O_2_-Ar mixture. Bruno et al. (2002) coupled Direct Simulation Monte Carlo (DSMC) with an StS kinetic model to study nonequilibrium processes in N_2_ behind strong shock waves, with particular attention to multi-quantum transitions and dissociation probabilities in V-T processes. Su et al. (2017) examined vibrational relaxation and chemical reactions in post-shock air flows and showed that NO formation is highly sensitive to the adopted reaction rate coefficients. Hong et al. (2019) compared the StS method with a classical two-temperature model for post-shock O_2_ flows and blunt-body stagnation-line flows, demonstrating that the StS approach predicts temperature and atomic O concentration distributions much more accurately than conventional two-temperature models. Gu et al. (2022) further investigated vibrational relaxation and NO formation in post-shock air flows and showed that multi-quantum transitions play an important role in the population of high-energy levels, whereas reducing V-V-T processes to V-T processes tends to overestimate vibrational excitation rates. Wang et al. (2025) further presented a direct benchmark comparison between StS and multi-temperature models for 11-species high-temperature air behind normal shock waves. Their results showed that the multi-temperature model predicts later internal-energy excitation and equilibration, and that the derived vibrational and electronic temperatures cannot accurately characterize the underlying nonequilibrium level populations. This work further confirms the advantage of StS models in describing strong post-shock nonequilibrium.

In addition to post-shock flows, StS models have also been widely applied to quasi-one-dimensional nonequilibrium nozzle flows relevant to high-enthalpy wind tunnels. High-enthalpy wind tunnels are important ground-based facilities for generating hypersonic flows, in which the test gas typically exhibits thermochemical nonequilibrium. Early StS studies of nozzle flows mainly focused on simple systems such as N_2_ and O_2_. Guy et al. (2013) investigated N_2_ nozzle flows and showed that the limitation on the maximum transition quantum number significantly affects the mole fraction and vibrational energy-level distribution of N_2_. Xu et al. (2014) studied quasi-one-dimensional nonequilibrium nozzle flow in N_2_ and found that the actual population distribution deviates markedly from the Boltzmann distribution characterized by a vibrational temperature.

For nonequilibrium nozzle flows of high-temperature air, studies usually consider five chemical species (N_2_, O_2_, NO, N, O). Early work often neglected the excited vibrational levels of NO. Colonna et al. (Colonna et al., 1998) applied an StS model to one-dimensional nozzle air flow, but only considered the vibrational levels of N_2_ and O_2_ while assuming NO to remain in its vibrational ground state. Nagnibeda et al. (Nagnibeda et al., 2018) also studied one-dimensional nozzle air flow using an StS model and compared the results with those from a one-temperature model; however, their treatment likewise assumed NO to be in the vibrational ground state. Wang H. et al. (2023) developed a more refined StS model by incorporating 48 vibrational levels of NO and showed that increasing the reservoir pressure helps reduce the degree of nonequilibrium in nozzle flows.

**FIGURE 4:**
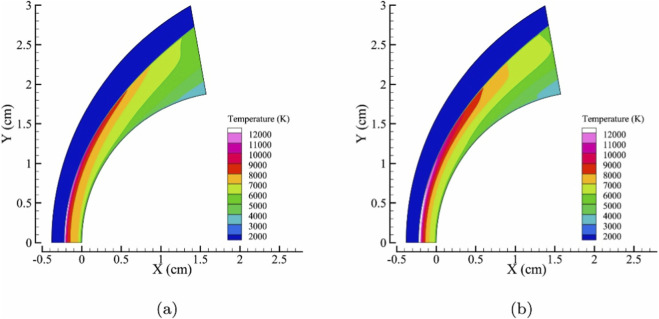
Gas temperature of a spherical body flow field calculated using different models: **(a)** StS model; **(b)** Park two-temperature model (Bonelli et al., 2024).

Building on these studies of canonical low-dimensional flows, StS models have gradually been extended to two-dimensional nonequilibrium flows and applied to specific high-enthalpy problems. Ninni et al. (Ninni et al., 2022a; 2022b) investigated two-dimensional double-wedge flows and axisymmetric double-cone flows using both multi-temperature and StS models, with particular attention to shock-wave/boundary-layer interactions and wall heat flux. Their results showed that the StS approach produced flow structures in closer agreement with experimental observations. Wang et al. (2023a) studied shock-wave/boundary-layer interactions in double-cone flows and found that the improvement provided by StS simulations was limited, suggesting that the discrepancies between experiments and simulations cannot be explained solely by deficiencies in thermochemical nonequilibrium modeling. Bonelli et al. (2024) extended air StS models to include CO kinetics and applied them to axisymmetric flow over a spherical body (Figure 4), demonstrating the potential of StS models for addressing ablation-related problems.

**FIGURE 5:**
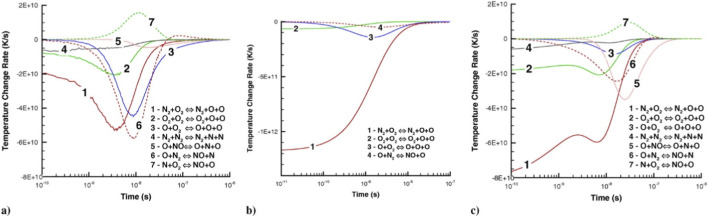
Impact of reaction channel on the rate of temperature change: **(a)** DSMC-M2 model (fully kinetic solver), **(b)** StS model (Vibrationally Kinetic Solver), **(c)** LeMANS-MMT model (continuum solver) (Gimelshein et al., 2022) reprinted by permission of the American Institute of Aeronautics and Astronautics, Inc.

Despite this progress, significant uncertainties remain in StS-based simulations of high-temperature air. Gimelshein et al. (2022) compared several fully kinetic, vibrationally kinetic, and continuum solvers for both adiabatic heat-bath conditions and hypersonic flow over a cylinder, and found noticeable discrepancies in the predicted vibrational relaxation times and reaction rate coefficients, as shown in Figure 5. These differences strongly affect the gas temperature and species mole fractions, indicating that current StS-based predictions remain sensitive to the adopted kinetic models and rate data. In particular, NO-related collisions remain one of the major sources of uncertainty, since the corresponding dissociation, recombination, and vibrational relaxation rates may differ by orders of magnitude across available studies. Such discrepancies are especially important because they directly affect the predicted NO peak behind the shock, the post-shock relaxation structure, and the surface heat-flux distribution.

Recent uncertainty-quantification studies on oxygen further show that the sensitivity of StS predictions to elementary kinetic data is not limited to NO chemistry. Wang et al. (2023c) performed Monte Carlo uncertainty and sensitivity analysis for state-specific modeling of thermal relaxation and dissociation of O_2_ behind a normal shock, treating 61,263 excitation and dissociation rates as uncertain parameters. Their results showed that uncertainties in the adopted rates can induce substantial variations in vibrational temperature and atomic O mole fraction, and that only a limited number of low-lying V-V-T and dissociation processes dominate the uncertainty of these macroscopic quantities. They also noted that even among QCT-based O_2_-O datasets, differences in the adopted potential energy surfaces may introduce nearly one-order-of-magnitude discrepancies in state-specific dissociation rates. These findings suggest that database reliability should be assessed not only from the underlying method used to generate the rates, but also from their sensitivity signatures in macroscopic predictions.

Compared with oxygen-related kinetics, for which relatively mature high-fidelity state-specific datasets and benchmark-oriented assessments have been reported (Andrienko and Boyd, 2015a; Neitzel, 2016), NO-related databases remain less consistent and less thoroughly validated (Gu et al., 2022; Su et al., 2017). Available validation strategies should therefore include not only microscopic calculations and database-level comparisons, but also hierarchical assessment against post-shock relaxation measurements, species distributions, radiation-relevant observables, and wall heat-flux predictions (Gimelshein et al., 2022; Gu et al., 2022; Su et al., 2017). In this context, systematic sensitivity analysis is especially valuable because it helps identify the small subset of elementary processes that controls the uncertainty of global thermochemical predictions (Aiken, 2023; Wang et al., 2023c).

### 3.2 Thermochemical nonequilibrium modeling from microscopic to macroscopic scales

Existing multi-temperature models are computationally efficient and therefore widely used in advanced CFD simulations. However, they cannot accurately represent non-Boltzmann energy distributions or capture vibrational-dissociation coupling, which limits their applicability and may lead to significant prediction errors. In contrast, StS models provide a more detailed description of energy-level distributions, but their application requires comprehensive and accurate rate coefficients for a vast number of elementary processes and entails high computational cost. For high-temperature air, the number of elementary StS processes can reach tens of millions, while the available rate coefficients are often incomplete or inaccurate. A practical way to address these challenges is to use microscopic distribution data from StS calculations to improve multi-temperature models, thereby enabling more accurate thermochemical nonequilibrium modeling while retaining computational efficiency.

**FIGURE 6:**
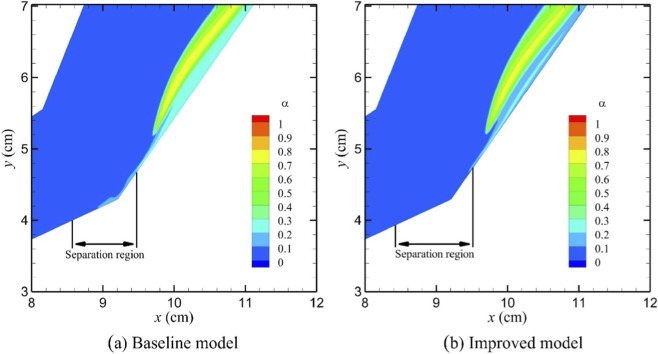
Dissociation degree of species O_2_ under different models: **(a)** Improved model; **(b)** Park two-temperature model (Hao and Wen, 2018).

Hao and Wen (2018) improved the CVDV model using kinetic rate coefficients for the V-V-T, V-T, and V-D processes of O_2_. Compared with conventional two-temperature models, the modified model predicted a larger separation zone in high-enthalpy double-cone flows and showed better agreement with experimental measurements (Figure 6). Mankodi and Myong (2019) proposed two new nonequilibrium models for fitting nonequilibrium reaction rate coefficients based on QCT calculations. The first, the nonequilibrium total temperature (NETT) model, introduces an additional term into the Arrhenius equation. The second, the nonequilibrium piecewise interpolation (NEPI) model, determines a set of fitting parameters for vibrational temperatures in the range of 1000-30,000 K. For vibrational temperatures outside this range, the parameters are obtained by linear interpolation. Both models are more accurate than the Park two-temperature model in simulating strongly nonequilibrium flows. Although these models improve the predictive accuracy of multi-temperature models through specific corrections, they do not account for non-Boltzmann distribution effects and therefore do not resolve the fundamental limitations of the multi-temperature framework. In a different approach, Neitzel et al. (Neitzel, 2016; Neitzel et al., 2017) modified the Park two-temperature model on the basis of StS simulations of O_2_ dissociation and proposed the two-temperature non-Boltzmann (2T-NENB) model, which shows close agreement with StS results. Chaudhry et al. (Chaudhry et al., 2020a; 2020b) introduced the Modified Marrone-Treanor (MMT) model, which incorporates non-Boltzmann corrections into the CVDV model to modify the nonequilibrium factor and the variation of vibrational energy. They applied both fixed and variable correction factors and calibrated the model using StS results. Inspired by the MMT correction approach, Wang et al. (2023b) introduced non-Boltzmann corrections into the Marrone-Fridman model and proposed the Modified Marrone-Fridman (MMF) model, whose reliability was validated against experimental measurements and StS results.

In addition to using StS results to refine multi-temperature models, coarse graining of vibrational levels provides another practical route for reducing the complexity of state-resolved kinetics. The coarse-grained model (CGM) simplifies the master equations by grouping energy levels according to the maximum-entropy principle (Liu et al., 2010; 2015) and prescribing the corresponding intragroup distribution pattern. Physically, the ansatz can be regarded as the least-biased reconstruction consistent with a limited set of retained macroscopic constraints, and it is therefore most appropriate when the detailed intragroup populations vary smoothly and can be represented by a small number of moments (Munafò et al., 2014; 2015). These resulting distributions can be represented using either linear (Liu et al., 2015; Magin et al., 2012; Munafò et al., 2014; 2015) or quadratic forms (Hao and Wen, 2019; Sharma et al., 2017; Sharma et al., 2020).

**FIGURE 7:**
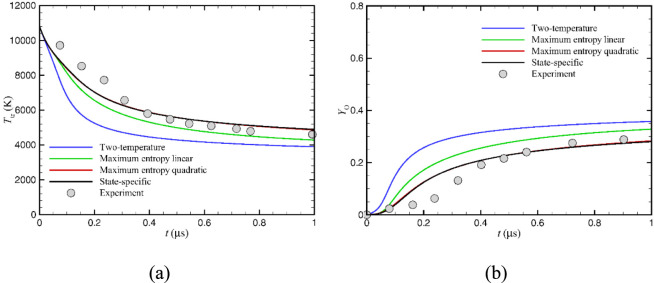
Results from different models under high-degree nonequilibrium conditions: **(a)** Translational-rotational temperature; **(b)** Atomic O mass fraction (Hao and Wen, 2019).

Under strongly nonequilibrium conditions, however, low-order maximum-entropy reconstructions may smooth out sharp non-Boltzmann tails and quasi-stationary structures near the dissociation continuum. As a result, linear multigroup formulations often require a large number of groups to recover the correct kinetics. To address this limitation, recent studies have focused on quadratic, piecewise-quadratic, and adaptive CGMs. Hao and Wen (2019) investigated O_2_ vibrational excitation and dissociation behind a shock wave using a two-temperature model, linear and quadratic maximum-entropy models, and StS simulations. Their results showed that, under strongly nonequilibrium conditions, the two-temperature model predicted atomic O mass fractions that differed significantly from experimental measurements, whereas the quadratic maximum-entropy model provided predictions closer to the experimental results than the linear model (Figure 7). Sharma et al. (2020) further showed that higher-order reconstructions can improve the representation of internal-energy distributions while reducing the number of groups. In addition, adaptive grouping can further improve local accuracy by adjusting the grouping according to the degree of nonequilibrium (Sahai et al., 2017).

Nevertheless, even these improved formulations do not guarantee accurate preservation of all strongly non-Boltzmann phenomena, especially when dissociation is controlled by a small number of highly excited states. Recent work has therefore also explored reduced-order strategies beyond conventional maximum-entropy closures. For example, Lv et al. (2024) developed a reduced-order model based on a CGM of vibrational states. By introducing a Treanor-like distribution together with an additional linear term, they overcame the limitations of a single-group CGM, which is equivalent to the two-temperature model. This enhancement enabled the model to more accurately capture the non-Boltzmann effects associated with highly excited vibrational levels. In addition, coupling the model with a fully connected neural network yielded an approximately 30-fold speedup. More recently, alternative reduced-order strategies beyond conventional maximum-entropy closures have also been proposed, highlighting that existing CGMs may remain inaccurate or insufficiently flexible in strongly nonequilibrium regimes (Zanardi et al., 2025). Overall, these developments suggest that coarse graining remains a promising route toward practical StS modeling, but its physical validity and resolution capability must be carefully assessed whenever high-energy tails and dissociation-controlling states play a dominant role.

## 4 Application of machine learning to state-to-state calculations

StS simulations can accurately resolve energy-level population distributions in hypersonic flows characterized by strong vibrational excitation and coupled chemical kinetics. However, the large number of required rate coefficients and the stiffness of the governing equations impose prohibitive computational and storage demands. Machine learning has therefore emerged as a promising route for accelerating StS calculations. Existing efforts can be broadly classified into two categories: (i) surrogate models for local kinetic or transport parameters and (ii) machine-learning-assisted solution of StS governing equations. Beyond this task-based classification, however, a more critical comparison is needed in terms of generalizability, data requirements, interpretability, and failure modes.

### 4.1 Machine learning for state-to-state key-parameter prediction

This direction focuses on replacing expensive local evaluations in StS simulations with machine-learning surrogates, thereby reducing the cost of repeated calls to rate, cross-section, or transport-property models. A major line of research concerns the prediction of kinetic parameters in StS models. Bushmakova and Kustova (2022) trained data-driven surrogates for relaxation rates in StS kinetic equations, using FHO- and SSH-based datasets, and showed that simple regressors can already provide useful accuracy in a 0D O_2_-O setting. Maksudova et al. (2024) further compared k-nearest neighbors, decision trees, and feed-forward neural networks for predicting StS dissociation rate coefficients of air species, showing that the neural-network model achieved the best accuracy among the tested approaches. Koner et al. (2019) combined importance sampling with residual neural networks to learn reaction cross sections from sparse QCT data, demonstrating that machine learning can provide efficient microscopic inputs for higher-level solvers. In addition, Istomin et al. (2023) applied neural networks to transport coefficients under strongly coupled vibrational and chemical nonequilibrium and reported speedups of approximately two orders of magnitude relative to conventional calculations.

Recent work has further expanded this line of research toward more direct prediction of state-to-state kinetic data. For example, Huang and Cheng (2024) reported machine-learning predictions of state-to-state dynamics for reactive and inelastic collisions relevant to hypersonic flows, while Mihalik et al. (2025) developed a probabilistic machine-learning framework for rate coefficients of state-to-state transitions in molecular collisions, with the explicit goal of accelerating calculations that would otherwise require expensive quantum scattering solutions. These developments indicate that machine learning is becoming increasingly relevant not only for interpolation within existing datasets but also for accelerating the generation of new state-specific kinetic data.

From a comparative perspective, local-parameter surrogates are attractive because they are relatively easy to integrate into existing CFD or reduced-order StS frameworks. Their main advantage lies in the separation between offline training and online deployment: once trained, they can dramatically reduce repeated local evaluations. However, their accuracy and generalizability depend strongly on the quality, coverage, and physical consistency of the labeled databases used for training. In particular, extrapolation to unseen species combinations, vibrational states, or temperature ranges remains a key risk. Moreover, interpretability varies substantially with the chosen model: simpler regressors may retain partial transparency, whereas deep neural models usually offer limited direct physical interpretability.

### 4.2 Machine learning for solving state-to-state master equations

Building on local-parameter prediction, recent studies have investigated machine-learning surrogates for directly solving StS master equations and Euler systems. The objective is to reduce computational cost by replacing or accelerating conventional numerical integration. Existing approaches can be broadly divided into physics-informed learning and purely data-driven modeling. In physics-informed approaches, governing equations or physical constraints are embedded into the learning process to improve consistency and robustness. Zanardi et al. (Zanardi et al., 2022; 2023) proposed a physics-informed Deep Operator Network (PI-DeepONet) for coarse-grained master equations and showed that it can learn the solution operator of the reduced kinetic system while bypassing traditional time integration. They later extended this framework through an adaptive, tree-based neural-operator architecture to capture multiscale vibrational relaxation and reported both improved accuracy and better efficiency in 0D and 1D settings.

Compared with physics-informed approaches, a larger body of work adopts purely data-driven strategies to predict state-resolved flow-field quantities or directly approximate the solution of StS governing equations. Campoli et al. (2022) identified relaxation-source-term evaluation and transport-model calculations as major computational bottlenecks in StS simulations and showed that deep neural networks can accelerate 1D reacting-shock calculations. Ozbenli et al. (2020) used artificial neural networks to replace direct numerical integration of the master equations and reported speedups of 2-3 orders of magnitude. Lv et al. (2023) proposed DeepStSNet, a data-assimilation framework coupling DeepONet with an StS coarse-grained model, thereby enabling the inference of vibrational quantum-state information from sparse macroscopic measurements.

More recent work has broadened this picture. Jacobsen et al. (2024) used information-theoretic machine learning to improve cluster assignment in coarse-grained nonequilibrium gas models, showing that data-driven learning can also enhance the reduced-order structure itself rather than only approximating rates or solutions. Kuppa et al. (2025) proposed stochastic operator learning for chemistry in nonequilibrium flows, with explicit attention to uncertainty representation, interpretability, and stable integration with fluid solvers. These developments suggest that the field is gradually moving beyond simple black-box surrogates toward hybrid approaches that combine machine learning with reduced-order modeling, uncertainty quantification, and physically constrained operator learning.

### 4.3 Critical comparison of machine-learning strategies for StS acceleration

Although the studies reviewed above demonstrate the promise of machine learning for accelerating StS calculations, their comparative strengths and limitations differ substantially. The most important distinction is not only the target task, but also the balance each method strikes among generalizability, data requirements, interpretability, and physical consistency.

In terms of generalizability, physics-informed approaches are generally more attractive because they embed governing equations or physical constraints into the learning process and are therefore less dependent on purely empirical correlations. This makes them potentially more robust when extrapolating across thermochemical states or initial conditions. However, such methods usually involve more difficult optimization and may still face challenges when applied to very stiff, multiscale, or multidimensional flows (Kuppa et al., 2025; Zanardi et al., 2023). By contrast, purely data-driven models can achieve remarkable speedups once trained, but their robustness outside the training domain often remains unclear. This issue is especially important in hypersonic nonequilibrium applications, where flow conditions may span wide ranges of temperature, pressure, and composition.

In terms of data requirements, local-parameter surrogates typically rely on high-quality labeled datasets for state-specific rates, cross sections, or transport coefficients. Their training data are expensive to generate, but once available, deployment is straightforward. Equation-solving surrogates usually require even more expensive reference data, including large numbers of solution trajectories or operator evaluations. Physics-informed approaches may reduce dependence on dense labeled datasets, but they shift the burden toward more complex training procedures and careful enforcement of physical constraints.

In terms of interpretability, machine-learning approaches cover a broad spectrum. Simple regressors and physically structured reduced-order models retain some degree of transparency, whereas neural operators and large deep networks are generally less interpretable unless additional physical structure is imposed. Recent studies on data-driven clustering and stochastic operator learning (Jacobsen et al., 2024; Kuppa et al., 2025) show that interpretability is becoming an increasingly explicit design objective, especially when machine learning is intended for integration into high-fidelity CFD workflows rather than merely offline acceleration.

Finally, the main failure modes of current machine-learning-assisted StS approaches include poor extrapolation to unseen thermochemical conditions, underrepresentation of highly excited states, loss of accuracy near strong gradients or shocks, and violation of physical consistency when conservation laws, positivity, equilibrium limits, or detailed balance are not explicitly enforced (Jacobsen et al., 2024; Kuppa et al., 2025; Zanardi et al., 2023). These limitations imply that the suitability of a given machine-learning method cannot be judged solely by in-sample error or speedup. Reliable deployment in future StS-CFD solvers will require broader validation across flow regimes, stronger physical guarantees, and closer integration with reduced-order and uncertainty-aware modeling strategies.

At present, no universal physical guarantees exist for purely data-driven machine-learning surrogates outside the training domain. The most effective safeguards are therefore partial rather than absolute. These include the enforcement of physical consistency constraints such as non-negativity, conservation laws, equilibrium limits, and, where relevant, detailed balance, as well as the use of physics-informed training, uncertainty-aware modeling, and broader out-of-distribution validation. Consequently, the robustness of machine-learning-assisted StS models should be assessed not only from in-domain prediction error, but also from their ability to preserve physically admissible behavior under unseen thermochemical conditions.

## 5 Summary and outlook

### 5.1 Summary

This review has provided a critical overview of recent advances and remaining challenges in vibrational state-to-state (StS) modeling for thermochemical nonequilibrium flows. Compared with traditional multi-temperature models, StS models provide a more physically rigorous description of high-temperature nonequilibrium gases by directly resolving state-specific energy transfer and chemical reactions, and they are therefore better suited to capturing non-Boltzmann effects and detailed vibration-dissociation coupling in hypersonic flows. Based on the discussions presented above, the main conclusions of this review can be summarized as follows.

1. The predictive capability of StS models depends fundamentally on the quality of elementary rate coefficients. The reliability of StS simulations depends strongly on the accuracy, consistency, and coverage of the underlying state-specific kinetic data. Existing rate coefficients are derived from methods with very different levels of physical fidelity and computational cost, ranging from empirical and semi-empirical models to trajectory-based and quantum-based approaches. Although high-fidelity methods have significantly improved the quality of available databases, substantial discrepancies still remain across datasets, especially for air chemistry under strongly nonequilibrium conditions. These inconsistencies constitute one of the main sources of uncertainty in StS predictions. This need is further reinforced by the fact that currently available experimental databases, although highly valuable, are still not sufficiently comprehensive to robustly validate StS models across the full range of realistic hypersonic nonequilibrium flows.
2. StS models have demonstrated clear advantages in canonical nonequilibrium flows, but their broader engineering deployment remains limited by several major numerical and practical barriers. Applications to post-shock flows, nonequilibrium nozzle flows, and selected two-dimensional high-enthalpy configurations have shown that StS models can improve the prediction of vibrational relaxation, dissociation, species distributions, and, in some cases, wall heat flux and flow-structure features. However, the routine use of full StS models in industrial three-dimensional CFD simulations is still hindered by the extremely large number of internal states and elementary processes, the strong stiffness of the governing equations, the high cost of Jacobian construction and implicit time integration, and the substantial memory and computational demands associated with large multidimensional grids.
3. Reduced-order strategies are essential, but their physical validity must be assessed carefully. Two major routes have emerged to alleviate the computational burden of StS modeling: the refinement of multi-temperature frameworks using StS-informed microscopic information, and the reduction of state-space complexity through coarse-grained models (CGMs). These approaches have achieved important progress, especially through non-Boltzmann corrections, maximum-entropy closures, adaptive grouping, and hybrid reduced-order formulations. Nevertheless, their performance depends on the validity of the adopted closures and reconstructions, and they may lose accuracy under strongly nonequilibrium conditions, particularly when highly excited states and dissociation-controlling populations play a dominant role.
4. Machine learning has become an important accelerator for StS modeling, but current progress remains uneven across tasks and methods. Recent studies have shown that machine learning can effectively accelerate the evaluation of state-specific rates, transport properties, and even reduced StS governing equations. However, the comparative advantages of different approaches depend strongly on the target task. Local-parameter surrogates are relatively easy to integrate into existing frameworks, whereas equation-solving surrogates can provide larger speedups but usually require more expensive training data and stronger safeguards against nonphysical behavior. Overall, machine learning has considerable potential, but its practical value depends not only on speedup and in-sample accuracy, but also on generalizability, interpretability, and physical consistency.
5. Uncertainty quantification and validation should be regarded as central components of future StS development. The review shows that disagreements among kinetic databases can propagate directly to macroscopic predictions, including vibrational temperature, species mole fractions, post-shock relaxation structure, and surface heat-flux distribution. This is particularly evident for air chemistry involving NO-related processes. Therefore, database assessment should not rely solely on the nominal fidelity of the underlying rate-generation method, but should also include sensitivity-guided and benchmark-oriented validation against experimental and macroscopic observables.

### 5.2 Outlook

Based on the analysis presented in this review, several directions deserve priority in future research on StS modeling for hypersonic thermochemical nonequilibrium flows.

1. Further progress in StS modeling requires more comprehensive and internally consistent databases of elementary rate coefficients. At present, many state-specific datasets are still assembled from heterogeneous sources with different theoretical assumptions, potential energy surfaces, fidelity levels, and applicable ranges. Future work should therefore place greater emphasis on systematic cross-comparison of available datasets, sensitivity analysis of key elementary processes, and uncertainty quantification linking microscopic rate coefficients to macroscopic aerodynamic and thermal predictions. Such efforts are essential for identifying the reactions that most strongly control prediction accuracy and for establishing more reliable validation standards.
2. Reduced-order modeling will remain indispensable if StS-based approaches are to be used more widely in practical CFD. Future reduced-order models should not only reduce computational cost, but also preserve the physically important nonequilibrium features that govern dissociation, recombination, and high-energy-state dynamics. This requires continued development of StS-informed multi-temperature corrections, improved CGMs, adaptive grouping strategies, and alternative projection-based or hybrid reduction methods. Particular attention should be paid to regimes in which conventional closures may fail, such as flows with strong non-Boltzmann tails or dominant quasi-stationary populations near the dissociation continuum.
3. Machine learning is likely to play an increasingly important role in future StS modeling, but the next stage of development should move beyond speedup alone. Greater emphasis should be placed on models that incorporate physical priors, conservation properties, equilibrium limits, and uncertainty awareness. In particular, improving out-of-distribution robustness, reducing dependence on expensive labeled datasets, and enhancing interpretability will be crucial if machine-learning-assisted StS models are to be trusted in demanding hypersonic environments. Hybrid frameworks that combine StS physics, reduced-order modeling, and machine learning appear especially promising.
4. In the long term, the most promising direction is not the unrestricted use of full StS models in industrial CFD, but the development of hybrid high-fidelity frameworks in which StS modeling provides physical guidance, benchmark data, and reduced-order closures for multidimensional simulations. Progress in this direction will require closer integration of kinetic-database development, reduced-order modeling, uncertainty quantification, machine learning, and experimental validation. Such an integrated strategy is likely to offer the most realistic path toward predictive and computationally tractable CFD tools for hypersonic aerothermodynamics and thermal-protection-system analysis.
